# Mitochondria-focused gene expression profile reveals common pathways and CPT1B dysregulation in both rodent stress model and human subjects with PTSD

**DOI:** 10.1038/tp.2015.65

**Published:** 2015-06-16

**Authors:** L Zhang, H Li, X Hu, D M Benedek, C S Fullerton, R D Forsten, J A Naifeh, X Li, H Wu, K N Benevides, T Le, S Smerin, D W Russell, R J Ursano

**Affiliations:** 1 Center for the Study of Traumatic Stress, Department of Psychiatry, Uniformed Services University of the Health Sciences, Bethesda, MD, USA; 2 U.S. Army Pacific Command, Hawaiian Islands, HI, USA

## Abstract

Posttraumatic stress disorder (PTSD), a trauma-related mental disorder, is associated with mitochondrial dysfunction in the brain. However, the biologic approach to identifying the mitochondria-focused genes underlying the pathogenesis of PTSD is still in its infancy. Previous research, using a human mitochondria-focused cDNA microarray (hMitChip3) found dysregulated mitochondria-focused genes present in postmortem brains of PTSD patients, indicating that those genes might be PTSD-related biomarkers. To further test this idea, this research examines profiles of mitochondria-focused gene expression in the stressed-rodent model (inescapable tail shock in rats), which shows characteristics of PTSD-like behaviors and also in the blood of subjects with PTSD. This study found that 34 mitochondria-focused genes being upregulated in stressed-rat amygdala. Ten common pathways, including fatty acid metabolism and peroxisome proliferator-activated receptors (PPAR) pathways were dysregulated in the amygdala of the stressed rats. Carnitine palmitoyltransferase 1B (CPT1B), an enzyme in the fatty acid metabolism and PPAR pathways, was significantly over-expressed in the amygdala (*P*<0.007) and in the blood (*P*<0.01) of stressed rats compared with non-stressed controls. In human subjects with (*n*=28) or without PTSD (*n*=31), significant over-expression of CPT1B in PTSD was also observed in the two common dysregulated pathways: fatty acid metabolism (*P*=0.0027, false discovery rate (FDR)=0.043) and PPAR (*P*=0.006, FDR=0.08). Quantitative real-time polymerase chain reaction validated the microarray findings and the CPT1B result. These findings indicate that blood can be used as a specimen in the search for PTSD biomarkers in fatty acid metabolism and PPAR pathways, and, in addition, that CPT1B may contribute to the pathology of PTSD.

## Introduction

Posttraumatic stress disorder (PTSD) is a complex mental disorder that can develop in response to a traumatic event (for example, an accident or combat exposure). In the United States, war-related PTSD carry a substantial social and financial burden considering there are approximately 500 000 veterans estimated to suffer from PTSD; as such, it is not surprising that the disease accounts for over 20 percent of all U.S. Department of Veterans Affairs compensation claims. However, little is known about the underlying molecular mechanisms of PTSD. Currently, a PTSD diagnosis is established on the basis of clinical history, mental health examination using a clinically structured interview and clinician administered or self-reported symptom checklist.^1^ The identification of biomarkers for risk of PTSD, PTSD presence or PTSD severity would help in the development of biologically based approaches to the assessment and management of the disorder.

In addition to neurochemical, functional and structural alterations in the neuroendocrine system in patients with such mental disorders, mitochondrial dysfunctions are increasingly recognized as key components in stress-related pathology.^2, 3, 4, 5^ The mitochondrion is a membrane-bound organelle present in most eukaryotic cells. It has a role in amino acid, lipid, steroid metabolism and apoptosis and acts as calcium buffer. It is also a source of free radicals.^6, 7^ The free radicals can damage mitochondrial DNA resulting in the production of more free radicals.^8^ Thus, the complementary functions of the mitochondria include bioenergetics, amino acid production and cell death.

Cumulative evidences from electron microscopy, imaging, gene expression, genotyping and sequencing studies indicate that there is mitochondrial dysfunction in schizophrenia, bipolar disorder, major depressive disorder and PTSD.^9, 10^ Monoamine oxidase A and B enzymes and benzodiazepine receptors, which are the psychopharmacological targets in the membrane of the mitochondria further supports the relationship between the mitochondria and psychiatric disorders.^11, 12, 13^ Therefore, direct examination of mitochondrial gene expression profiles from animal models and subjects with PTSD may provide further details of the role of mitochondria in psychiatric disorders in general, and PTSD in particular, and help identify possible biomarkers for PTSD.^4^

Previous research has demonstrated mitochondrial dysfunction in the PTSD brain cortex, indicating that expression fingerprints may serve as biomarkers for a PTSD diagnosis.^4^ This research found PTSD-specific expression fingerprints of 800 informative mitochondria-focused genes across all 12 of the postmortem brains sampled, and 119 (±>1.25, *P*<0.05) and 42 (±>1.60, *P*<0.05) dysregulated genes between the PTSD and control samples.^4^ These fingerprints distinguished the PTSD cortices from controls. The 119 dysregulated genes were associated with mitochondrial dysfunction, oxidative phosphorylation, cell survival apoptosis and neurological diseases. Fifty dysregulated genes were present in the molecular networks known to be involved in neuronal function survival and that contain targets for neuropsychiatric drugs.^14^ Thirty of these dysregulated mitochondria-focused genes are associated with a number of neuropsychiatric disorders. These results are consistent with others showing the relationship between traumatic stress and mitochondrial dysfunction.^2, 3^ For example, psychological stress and anxious behavior^15^ are associated with oxidative stress;^16^ the enzymes involved in oxidative stress (glyoxalase 1 and glutathione reductase 1) regulate anxiety in mice.^15^ Chronic stress inhibits the activity of mitochondrial respiratory complexes in rats.^17^ The stress hormone cortisol results in the binding of the glucocorticoid receptor to mitochondrial membranes and regulates apoptosis.^2^ In this study, we examined profiles of mitochondria-focused gene expression in the stressed-rodent model (inescapable tail shock in rats), using rat mitochondria neuronal chip containing 37 mitochondrial DNA-encoded genes, 1,098 nuclear DNA-encoded and mitochondria-focused genes and 365 genes associated with neuronal functions. The inescapable tail shock rat model has shown PTSD-like behaviors and also biochemical changes seen in subjects with PTSD.^18, 19^ Utilizing an identical human mitochondria-focused gene microarray (hMitChip3) approach in conjunction with our previous human brain data, we examined whether the profile of stress-induced gene expression in animals was also present in the blood of subjects with PTSD and therefore whether blood can be used in the search for mitochondria-related PTSD biomarkers.

## Materials and methods

### Stressed-animal model

Male albino Sprague Dawley rats were used (Taconic Farms, Derwood, MD, USA), weighing 150–200 g at the time of administration of the stress protocol. Two groups of animals were studied: Group 1 (*n*=10) received the stress protocol and Group 2 (*n*=10) served as the control. Stress exposure consisted of a daily 2-h session of restraint by immobilization in ventilated Plexiglas tubes (inescapable) and tail shocks for three consecutive days. Stressing was performed each morning between 0800 and 1200 h. Forty electric shocks (2 mA, 3-s duration; Animal Test Cage Grid Floor Shocker, Coulbourn Instruments, Whitehall, PA, USA) were delivered to the tail of Group 1 rats at semi-random intervals of 150–210 s (Graphic State Notation software, Habitest Universal Link, Coulbourn Instruments). Electrode gel was applied using Q-tips to form a thin layer of conducting gel between the electrode and the skin of the rat's tail. The electrode clips were adjusted and connected to the tail to ensure a good connection without affecting the blood circulation of the tail.

Amygdala dissection was performed as previously described in stressed-animal model.^18^ In this animal model, the stressed rats exhibited: (1) a delayed and exaggerated startle response appearing several days after stressor cessation, which, given the compressed time scale of the rat's life compared with that of a human, corresponds to the 1–3-month delay of symptoms in PTSD patients;^20^ (2) enhanced plasma corticosterone for several days, indicating a compromised hypothalamopituitary axis; and (3) retarded body weight gain after stressor cessation, indicating dysfunction of gene expression. The gene expression microarray used in this experiment, dubbed the rat mitochondria neuronal chip, focuses on mitochondrial and mitochondria-related nuclear genes.

### Rat mitochondria neuronal chip

The rat mitochondrion-neuron focused microarray was designed and fabricated by GenProMarkers (Rockville, MD, USA). The rat mitochondria neuronal chip contains 1500 genes, including 37 mitochondrial DNA-encoded genes, 1098 nuclear DNA-encoded and mitochondria-focused genes and 365 genes associated with neuronal functions, including fear response, circadian rhythms, intraneuronal signal transduction and neurotransmitters. The oligonucleotides used were designed with the software MacVector v10.6.0 (MacVector) using full-length messenger RNA (mRNA) sequences as the templates according to published criteria. An amino-C6 modifier was added to the 5′ end of each oligonucleotide probe to enhance the binding of the DNA to glass slides and the accessibility of hybridization with target DNA. The 1500 test genes (including 80 ‘housekeeping' genes as positive controls) and 36 negative controls (non-rat DNA) were printed, each in triplicates, onto the *N*-hydroxysuccinimide ester reactive group-coated glass slides (CodeLink Activated Slide, SurModics, Eden Prairie, MN, USA). DNA probes in the print buffer (50 mM sodium phosphate), at a final concentration of 20 μM of 5′-amino-C6 modified 50-mers, were printed in the Class 100 super-clean environment, using 100-micron pins and the GeneMachine OmniGrid 100 Microarrayer (Genomic Solutions, Ann Arbor, MI, USA).

### Rat RNA purification

The amygdalae of rats and whole blood from both the stress (*n*=10) and control (*n*=10) groups were dissected immediately after euthanasia.^18^ Total amygdala RNA was purified from rat brain tissues by using a PAXgene RNA Kit (QIAGEN, Valencia, CA, USA) following the manufacturer's instructions.

### Microarray hybridization for rat study

One microgram of RNA per sample was used for Cy5-dUTP labeling of cDNA by use of the expression array detection kit (3DNA Array 900, Genisphere, Hatfield, PA, USA) following the manufacturer's instructions. Slides were scanned using 5-micron resolution and the LOWESS method with a ScanArray Microarray Scanner (PerkinElmer, Waltham, MA, USA). Triplicate microarray experiments were performed for each RNA sample purified from the amygdala and human blood. The background-subtracted mean values of the measured gene expression signal intensities were used for microarray data analysis. All microarray experiments were performed in the same laboratory (GenProMarkers).

#### Microarray database, bioinformatics and systems biology for rat study

A gene expression database was constructed using FileMaker software (FileMaker Pro, Santa Clara, CA, USA). Database construction, data filtering, selection, exclusion and inclusion procedures and criteria were performed as described previously (see below human microarray procedure). The quintile normalization method,^21, 22^ in software R version 2.15.1 (R Foundation for Statistical Computing) was used to normalize background-subtracted mean intensities across all intra- and inter-slides of informative microarray data. The normalized data were used to cluster and visualize genes and samples using Cluster version 3.0.

The resulting heat map was visualized by using MapleTree software (http://rana.lbl.gov/EisenSoftware.htm). The normalized data were also used for the calculation of means, standard deviations, fold changes, moderated *P-*values and false discovery rates (FDRs). Gene IDs, official symbols and official full names were updated using the NCBI database (www.ncbi.nlm.nih.gov/gene). Kyoto Encyclopedia of Genes and Genomes pathways and Online Mendelian Inheritance in Man were from DAVID Bioinformatics Resources (http://david.abcc.ncifcrf.gov).

### Human subjects

Subjects with (*n*=28) or without (*n*=31) PTSD were sampled from active duty U.S. Army Special Operations Command units. The protocol was approved by the Institutional Review Board of the Uniformed Services University. All participants provided written informed consent for the study. Current PTSD symptoms were assessed using a summative score derived from the DSM-based PTSD checklist, which is a 17-item self-report validated measure. A probable PTSD diagnosis was made if subjects endorsed DSM-IV PTSD criteria (at least one re-experiencing, three avoidance and two hyper-arousal symptoms) and had a total PTSD checklist score of 50 or above. Subjects' blood mRNA were used for microarray and quantitative real-time polymerase chain reaction (qRT-PCR).

### hMitChip3

A third-generation hMitChip3 containing all 37 mitochondrial DNA-encoded genes, 1098 nuclear DNA-encoded and mitochondria-related genes and 225 controls were printed as described previously.^4^ A total of 1135 mitochondria-related genes were sampled, including 946 genes associated with 645 molecular functions, 930 genes with 612 biological processes, 476 genes with biochemical pathways, 227 genes with 23 reactome events, 237 genes with 320 genetic disorders and 55 genes with 87 drugs targets.^4^ For example, 89 genes are related to oxidative phosphorylation pathways. Each gene on the hMitChip3 gene chip was printed in triplicate onto polylysine-coated glass slides in the Class 100 super-clean environment as established in the literature using 100-micron pins and the GeneMachine OmniGrid 100 Microarrayer (Genomic Solutions).^23^

### RNA purification of human subjects

Each sample of total RNA was purified from 2.5 ml of human peripheral blood collected in a PAXgene blood RNA tube (Catalog No. 8634, PreAnalytiX) and processed using the PAXgene Blood RNA Kit (Catalog No. 762164, PreAnalytiX) following the manufacturer's instructions.

### Microarray labeling and hybridization, image scanning and processing

One microgram RNA per sample was used for Cy5-dUTP (Enzo Life Sciences, Plymouth Meeting, PA, USA) labeling of cDNA. The cDNA synthesis and microarray hybridization were carried out using an expression array detection kit (3DNA Array 900, Genisphere, Hatfield, PA, USA) following the manufacturer's instructions. Slides were scanned using the LOWESS method in the ScanArray Microarray Scanner (PerkinElmer) at 90% laser power, 68 PMT voltages and 5-micron resolution.

Microarray images were quantified by use of the hMitChip3 gene array list file. In the digitized data, each scanned spot was labeled by a flag either as ‘0' (found but not good), ‘1' (not found), ‘2' (absent), ‘3' (good) or ‘4' (bad). The ‘good' spots were defined by the scanner software setups as the spot with a calculated footprint <100 μm. Triplicate microarray experiments were performed for each and every RNA samples. Due to the presence of both technical triplicates and experimental triplicates, the RNA level of each and every hMitChip3 gene was measured nine times. The background-subtracted mean values of the measurements were used for microarray data analysis. All microarray experiments were performed in the same GenProMarkers laboratory.

### Microarray database and data analysis

A gene expression database was constructed using FileMaker software (FileMaker Pro). Database construction, data filtering and selection were performed as described previously.^23^ The quintile normalization method^3, 4^ in software R version 2.7.1 (R Foundation for Statistical Computing) was used to normalize microarray data. The normalized expression data were used to cluster and visualize genes and cell lines by using software Cluster version 3.0 7 and the resulting heat map was visualized by using software MapleTree (http://rana.lbl.gov/EisenSoftware.htm). The ID, official symbols and full names of genes on hMitChip3 were updated to human UniGene Build 219 (ftp://ftp.ncbi.nih.gov/repository/UniGene, October 2009) based on human cDNA I.M.A.G.E. clone ID (http://image.llnl.gov/).

### Quantitative RT-PCR

One microgram of total RNA was reverse transcribed into cDNA by using SuperScript First-Strand Synthesis System (Invitrogen). Thirty nanograms of cDNA was used for qPCR reactions on an Applied Biosystems 7900 HT Real Time PCR System (Foster City, CA, USA), following the manufacturer's instructions. After 40 cycles, data reduction was performed with the 7900 HT PCR Software. Duplicate qRT-PCR experiments were performed for carnitine palmitoyltransferase 1B (CPT1B) gene. Relative RNA concentrations were calculated using the published methods.^24^ TaqMan probe and primers for qPCR were purchased from Applied Biosystems and included β-actin and CPT1B (Life Technologies, Grand Island, NY, USA).

### Statistics

The quintile normalization method in software R/Bioconductor version 2.15.1 (The R Foundation for Statistical Computing) was used to normalize data. Means, standard deviations and fold changes were calculated from triplicate spots and triplicate experiments using XLSTAT 2006 (XLSTAT, New York, NY, USA). Differentially expressed genes were arbitrarily identified as having a ⩾2-fold change in the average expression of the background-subtracted mean intensity ratios of a gene between comparisons.^25^ The moderated *P*-values and FDR for multiple statistical testing with Benjamini and Hochberg methods^26^ were calculated with the software R/Bioconductor version 2.15.1 (The R Foundation for Statistical Computing). Differentially expressed genes were identified arbitrarily by ⩾1.25-fold change in the average expression of the background-subtracted mean intensity ratios of a gene between PTSD and control in microarray experiment, controls and PTSD in qPCR assay, as well as stressed and non-stressed rats with *P*-value <0.05. Student *t*-test was used to calculate *P*-values for gene expression, while Fisher exact test in Ingenuity Pathway Analysis software was used to calculate *P*-values for pathways and diseases. The level of statistical significance was set at a *P*-value <0.05.

## Results

**Figure 1:**
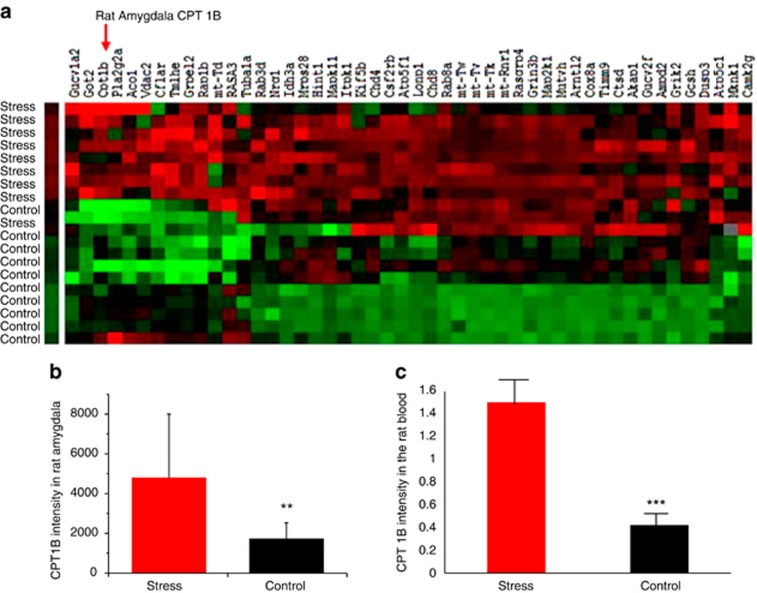
Dysregulation of CPT1B in stressed-rodent model. (**a**) Dendrogram and heat maps of the expression of genes in amygdala of stressed (*n*=10) and non-stressed (*n*=10) rats. (**b**) Stress-induced overexpression of CPT1B in the rat amygdala. (**c**) Stress-induced overexpression of CPT1B in the rat blood. **P<0.01, ***P<0.001. CPT1B, carnitine palmitoyltransferase 1B.

We first quantified the scanned image into numeric data amenable to statistical analysis. The raw data set was filtered over 610 740 spots across all 174 gene chips of 20 rats (stressed and non-stressed) and 59 human subjects (PTSD and non-PTSD) by removing high-noise and low-signal spots. Data were normalized to remove spatial variability, channel imbalances and inter-array heterogeneity. Cluster approaches were next used to identify broad patterns. Using this approach, we were able to produce a visualization of the data as a hierarchical clustering. The hierarchical clustering generated dendrograms demonstrating up- and downregulated gene clusters in amygdala of rats with or without stress (Figure 1a) and subjects with or without PTSD. These unsupervised data indicated grouping of genes having similar expression patterns or clustering. The approaches are based on the assumption that the whole set of microarray data is a finite mixture of a certain type of distribution with different parameters. Stress-induced upregulated genes in rat's amygdala are listed in Table 1.

In the stressed-rodent model, we found that the stress resulted in 34 genes being upregulated (Table 1 and Figure 1a, in red) in the amygdala complex by stringent criteria. A high stringency algorithm was used, including fold changes and FDR comparing stressed to controls (Table 1). The tables include gene symbols, full names of genes listed in Genecards, fold change (ratio of stress and control, log(2) of the ratio), *P*-value and FDR. Among those mitochondria-related gene(s), CPT1B, an enzyme in the fatty acid metabolism and peroxisome proliferator-activated receptors (PPAR) pathways, was significantly dysregulated. CPT1B was significantly overexpressed in the amygdala of stressed rats compared with non-stressed controls (*P*<0.007, FDR=0.013, Figure 1b). Consistent with the stress-induced changes in the amygdala, CPT1B mRNA in the blood (Figure 1c) was also significantly over-expressed in the stressed rats compared with non-stressed controls (*P*<0.001).

**Figure 2:**
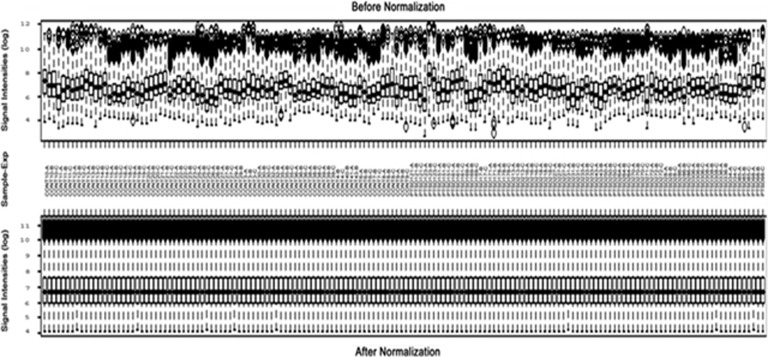
Box plots of expression data before and after normalization. The quintile normalization algorithms were used to adjust the values of the background-subtracted mean pixel intensities of each and every set of 800 genes that were selected from the hMitChip3 triplicate microarray experiments hybridized with PTSD and control prefrontal cortex RNA samples, as described previously.^4^ In contrast to the pre-normalization boxplots (top panel), the post-normalized boxplots distribute in the same intervals with the same density center, indicating successful adjustment of data. The post-normalized data were used for clustering analysis. PTSD, posttraumatic stress disorder.

**Figure 3:**
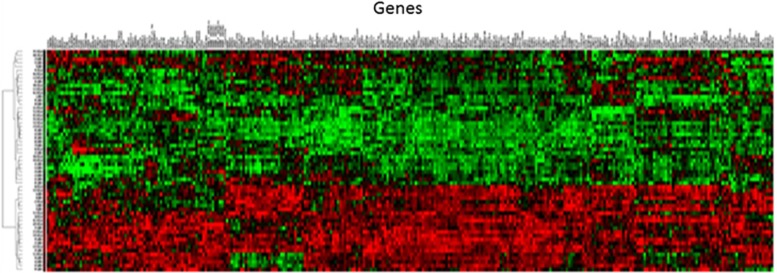
Dendrogram and heat maps of the expression of 308 downregulated genes in the blood cells of subjects with PTSD (*n*=28) and control subjects (*n*=31). PTSD, posttraumatic stress disorder.

**Figure 4:**
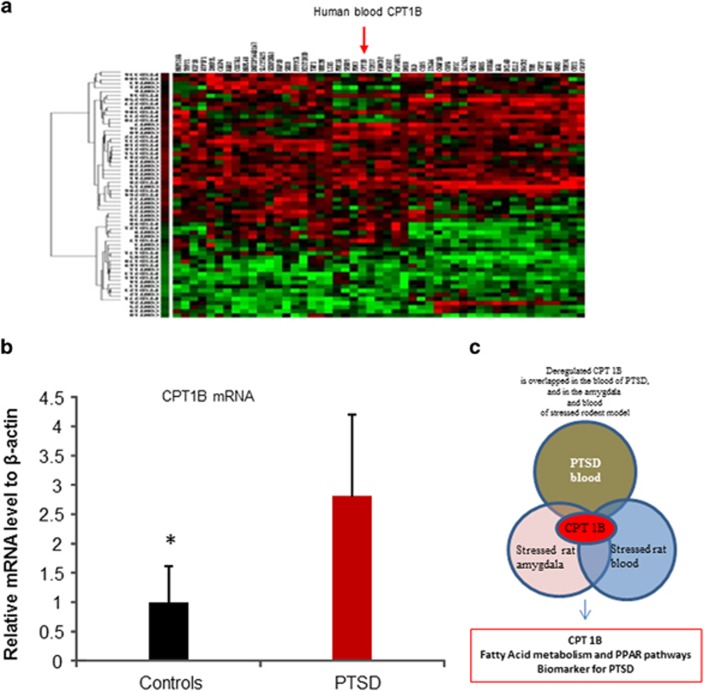
(**a**) Dendrogram and heat maps of the expression of upregulated genes including CPT1B and qRT-PCR data of CPT1B in the blood of subjects with (*n*=28) and without PTSD (*n*=31). (**b**) CPT1B mRNA level was significantly higher in subjects with PTSD than that in the non-PTSD controls (*P<0.05). (**c**) Diagram presenting the model of overexpression of CPT1B in subjects with PTSD and stressed animals. CPT1B, carnitine palmitoyltransferase 1B; mRNA, messenger RNA; PTSD, posttraumatic stress disorder; qRT-PCR, quantitative real-time polymerase chain reaction.

In human subjects, total RNA samples were extracted from blood of subjects with or without PTSD and labeled for triplicate microarray experiments using our recently developed third-generation microarray hMitChip3. Table 2 shows the diagrammatic information about the subjects with PTSD and without PTSD, including their age, sex and ethnicity. To avoid misclassification, the hMitChip3 genes were all measured nine times (three identical probes per microarray and three microarray experiments per specimen), which generated reliable expression data for further analysis.^4^ The microarray data of 610 740 spots across all 531 gene chips used for 59 RNA samples were filtered by uniform statistic and bioinformatic criteria described previously,^27, 28^ which generated 591 genes with informative expression profiles. Figure 2 shows the boxplots of mRNA levels of 1170 genes before and after the data normalization. The normalized data were used for unsupervised clustering analysis and visualization (Figure 3). The resultant dendrograms for the sets of genes are indicated in Figures 3 and 4.

On the basis of the unsupervised cluster results, the analytic approach of unsupervised pattern recognition and Kyoto Encyclopedia of Genes and Genomes revealed 10 clusters or pathways (Table 3). These pathways are involved in several networks related to neuronal disorders, such as Alzheimer's disorder, Huntington and Parkinson's disease, fatty acid metabolism and PPAR (Table 3). Again CPT1B (Figure 4a) was observed in the two common dysregulated pathways (Table 3): fatty acid metabolism (*P*=0.0027, FDR=0.043) and PPAR (*P*=0.006, FDR=0.08). The qRT-PCR validated the microarray result, showing that CPT1B mRNA was significantly overexpressed in subjects with PTSD than in the non-PTSD controls (Figure 4b).

## Discussion

The aim of this study was to examine the expression profiling of mitochondria-focused genes in the blood of stressed rats and PTSD patients and to identify gene clusters, common pathways and signal genes related to PTSD in both a stressed-animal model of PTSD and human subjects with PTSD. Animal models of PTSD offer opportunities to identify potential biomarkers for this disorder. In this study, we demonstrated that a substantial number of up- and downregulated genes related to mitochondrial function in the amygdala are associated with an animal model of PTSD. These mitochondria-focused genes have been previously related to neurological dysfunction, psychiatric disorders and exaggerated fear response. In addition, we identified novel mitochondria-focused genes associated with fatty acid metabolism and PPAR signaling, specifically in relation to CPT1B in human blood.

In our previous work, we utilized high-throughput genomics and mitochondria-focused gene microarrays, which have informed an unprecedented surge of cross-disciplinary work between biology and statistics.^4^ For microarray, quality assessment of data is an important yet often challenging aspect of gene expression analysis. The application of machine-learning techniques (also known as pattern-recognition techniques) to large biological data sets has been used to identify groups of functionally related genes,^29^ to predict broader biological phenotypes and genetic interactions^30^ and to diagnosis PTSD.^4^ We found that there were 10 pathways in the groups of upregulated genes in human blood. These clusters of dysregulated mitochondria-focused genes are involved in neuron function and neurological disorders, such as Alzheimer's disorder, Huntington and Parkinson's diseases, as well as the metabolic and signaling pathways. Networks and pathways might be central to drug development for PTSD and patient management tools.

Biomarkers are classically viewed as individual genetic or protein variants associated with disease risk, diagnosis, severity or course. In so complex a disorder as PTSD, clustering a variety of risk factors (Table 3) can improve predictive value. Integrative biomarkers can be developed through superposition of mRNA. Clustering can prioritize functionally plausible candidate genes.^31^ Clustering aggregates information from multiple loci into association scores for pathways^32^ and informs drug development by predicting therapeutic gene targets. Clustering can also identify pathways affected by an existing drug, either to predict the mode of action or suggest drug repurposing in which drugs developed for known disorders can serve as a new compound for PTSD. We found that CPT1B in the fatty acid metabolism and PPAR pathways was significantly over-expressed, not only in the amygdala (Figures 1a and b) but also in the blood of stressed rats (Figure 1c). These data suggest that fatty acid metabolism and PPAR pathways, and CPT1B were dysregulated at the transcription level in brain and in the peripheral blood of the rats exposed to the stress.

We also examined whether the dysregulated gene pathways and CPT1B seen in the stressed-rat model could be observed in human subjects. To accomplish this, we accomplished a profiling of mitochondria-focused genes using blood samples from subjects with and without PTSD using hMitChip3 microarray. Ten dysregulated clusters or pathways were identified, including fatty acid metabolism and PPAR (Table 3). Again CPT1B was observed in the two common dysregulated pathways: fatty acid metabolism and PPAR (Table 3 and Figure 4a). In our RT-PCR confirmation experiment, we found that CPT1B mRNA was significantly overexpressed in subjects with PTSD compared with non-PTSD controls (Figure 4b).

Fatty acids are a family of molecules in the lipid macronutrient class.^33^ One of their roles is energy production in the form of adenosine triphosphate synthesis. Compared with carbohydrates and protein, fatty acids yield the most adenosine triphosphate on an energy per gram basis. Fatty acid metabolism also has other roles including energy storage, phospholipid membrane formation and signaling. Previously, direct evidence has shown that fatty acid-induced gut-brain signaling attenuates neural and behavioral effects of sad emotion in humans.^34^ Fatty acids are PPAR ligands, which activate PPAR receptors and increase target gene transcription.^35^ The PPAR pathway regulates lipid metabolism, cellular differentiation and proliferation. Downregulation of PPAR may result in metabolic syndrome-related disorders, such as insulin resistance and hypercholesterolemia. These data indicate that genes (for example, CPT1B) mainly associated with the fatty acid metabolic pathway may have a role in PTSD. Currently, there is little information about the roles of the common pathways (that is, fatty acid metabolism and PPAR signaling) in relation to PTSD.

Altogether, utilizing a mitochondria-focused gene microarray approach in conjunction with our previous human brain data,^4^ the results from these three independent experiments (stressed-rat amygdala, stressed-rat blood and PTSD subject blood) support the hypothesis that dysregulation of mitochondria-focused gene(s),^2^ specifically CPT1B in the fatty acid metabolism and PPAR pathways, may be a part of the pathology of PTSD (Figure 4c). Furthermore, these findings suggest the use of blood in the search for PTSD biomarkers and indicate possible novel therapeutic avenues for the treatment and management of PTSD.

## Figures and Tables

**Table 1:** Upregulated genes in stressed rat amygdala

*Gene*	*Names of genes listed in gene cards*	*Fold changes*		*Significance*	
		*Stress/Con*	*Log(2)*	P*-value*	*FDR*
*Akap1*	A kinase anchor protein 1	2.39	1.25	0.0098	0.0155
*Arntl2*	Aryl hydrocarbon receptor nuclear translocator-like 2	7.86	2.97	0.0001	0.0028
*Atp5c1*	ATP synthase, H+ transporting, mitochondrial F1 complex, gamma polypeptide 11	2.36	1.24	0.0111	0.0165
*Atp5f1*	ATP synthase, H+ transporting, mitochondrial Fo complex, subunit B1	3.02	1.59	0.0002	0.0029
*Camk2g*	calcium/calmodulin-dependent protein kinase II gamma	4.13	2.05	0.0032	0.0086
*Cox8a*	cytochrome c oxidase subunit VIIIA (ubiquitous)	2.61	1.38	0.0024	0.0071
*CPT1B*	carnitine palmitoyltransferase 1B (muscle)	2.80	1.49	0.0074	0.0133
*Csf2rb*	colony stimulating factor 2 receptor, beta, low-affinity	6.11	2.61	0.0005	0.0044
*Ctsd*	cathepsin D	3.30	1.72	0.0017	0.0055
*Dusp3*	Dual specificity protein phosphatase 3	2.03	1.02	0.0107	0.0163
*Got2*	Glutamic-oxaloacetic transaminase 2	2.27	1.18	0.0077	0.0133
*Grik2*	Glutamate receptor, ionotropic, kainate 2	2.06	1.04	0.0012	0.0047
*Grin3b*	Glutamate receptor, ionotropic, N-methyl-D-aspartate 3B protein	4.77	2.25	0.0016	0.0055
*Grpel2*	GrpE-like 2, mitochondrial (*E. coli*)	13.4	3.75	0.0009	0.0047
*Gucy1a2*	Guanylate Cyclase 1, Soluble, Alpha 21	2.05	1.03	0.0023	0.0071
*Gucy2f*	Guanylate cyclase 2F, retinal	2.34	1.23	0.0075	0.0133
*Idh3a*	Isocitrate dehydrogenase 3 (NAD+) alpha	2.57	1.36	0.0034	0.0071
*Itpk1*	Inositol 1,3,4-trisphosphate 5/6 kinase2	5.59	2.48	0.0006	0.0044
*Kif5b*	Kinesin family member 5B	3.21	1.68	0.0147	0.0199
*Lonp1*	Lon peptidase 1, mitochondrial	12.0	3.59	0.0016	0.0086
*Mrps28*	Mitochondrial ribosomal protein S28	2.21	1.14	0.0143	0.0199
*mt-Rnr1*	Mitochondrially encoded 12S RNA1	4.05	2.02	0.0001	0.0024
*mt-Td*	Mitochondrially encoded tRNA aspartic acid	14.2	3.82	0.003	0.0086
*mt-Tk*	Mitochondrially encoded tRNA lysine	3.69	1.88	0.0006	0.0044
*mt-Tw*	Mitochondrially encoded tRNA tryptophan	3.18	1.67	0.0011	0.0047
*mt-Ty*	Mitochondrially encoded tRNA tyrosine	3.44	1.78	0.0011	0.0047
*Mutyh*	Muty homolog (*E. coli*)	7.92	2.99	0.0141	0.0199
*Nrg1*	Neuregulin 1	3.34	1.74	0.0006	0.0044
*Rap1b*	Ras family small GTP binding protein RAP1B2	10.1	3.34	0.0014	0.0053
*Rab8a*	Member RAS oncogene family	3.54	1.82	0.0000	0.0014
*RASA3*	Ras GTPase-activating protein 3	2.02	1.01	0.0040	0.0091
*Rasgrp4*	RAS guanyl releasing protein 4	3.62	1.86	0.0008	0.0047
*Timm9*	Translocase of inner mitochondrial membrane 9 homolog	3.49	1.80	0.0011	0.0047
*Tmlhe*	Trimethyllysine hydroxylase, epsilon	6.71	2.75	0.0007	0.0045

Abbreviations: ATP, adenosine triphosphate; FDR, false-discovery rate; tRNA, transfer RNA.^36^

**Table 2:** Demographic information

	*Controls*	*PTSD*	P-*value*
Age	21.4±5.0	27.5±5.0	0.002
			
*Sex*			0.80
Male	28	23	0.05
Female	4	4	
Unknown	0	1	
			
*Ethnic*			0.32
AAN	0	0	
API	3	3	
Black	3	6	
White	26	21	

Abbreviations: AAN, American Indian or Alaskan Native; API, Asian or Pacific Islander; PTSD, posttraumatic stress disorder.

**Table 3:** Pathways with significant number of genes with altered expression

	*KEGG pathway*	*Genes with alternated expression*	P*	*FDR*
1	Oxidative phosphorylation	ATP5F-1, ATP5G1, ATP5G2, ATPG3, ATP5L, ATP5H, ATP5O, ATP5B, COX5A,COX7A1, COX10, COX11, NDUFA3, NDUFA4, NDFA9, NDUFB4, NDUFC1, NDUFS2, NDUFS5, NDUFS8, NDUFV2, A2,SDHD,UQCRC2, UQCRH	2.0E−15	3.4E−13
2	Alzheimer's disease	ATP5B, ATP5F-1, ATP5G1, ATP5G2, ATPG3, ATP5H, ATP5O, CALM2, CASP7,COX5A, COX7A1, NDUFA3, NDUFA4, NDFA9, NDUFB4, NDUFC1, NDUFS2, NDUFS5, NDUFS8, NDUFV2, NOS1, PSEN1, SDHD, UQCRC2, UQCRH	5.2E−13	3.3E−11
3	Huntington's disease	ATP5B, ATP5F-1, ATP5G1, ATP5G2, ATPG3, ATP5H, ATP5O, BBC3, COX5A, COX7A1, NDUFA3, NDUFA4, NDFA9, NDUFB4, NDUFC1, NDUFS2, NDUFS5, NDUFS8, NDUFV2, SDHD, SOD1, UQCRC2, UQCRH, VDAC1	6.3E−13	2.7E−11
4	Parkinson's disease	ATP5B, ATP5F-1, ATP5G1, ATP5G2, ATPG3, ATP5H, ATP5O, COX5A, COX7A1, NDUFA3, NDUFA4, NDFA9, NDUFB4, NDUFC1, NDUFS2, NDUFS5, NDUFS8, NDUFV2, SDHD, UQCRC2, UQCRH, VDAC1	2.0E−12	6.5E−11
5	Glycolysis/Gluconeogenesis	ALDH, ALDH3B1, ALDH3B2, ALDH7A1, ALDOA, ALDOC, DLD, HK1, LDHB, PDHA1, PGAM2, TPI1	1.6E−7	4.0E−6
6	Pyruvate metabolism	ACACB, AKR1B1, ALDH2, ALDH7A1, DLD, HAGH, LDHB, PDHA1	4.4E−5	9.2E−4
7	Fatty acid metabolism	ACADM, ACADVL, ACSL5, ALDH2, ALDH7A1, CPT1B	2.7E−3	4.3E−2
8	Citrate cycle (TCA cycle)	ACLY, DLD, PDHA1, SDHD, SUCLG1	6.6E−3	8.1E−2
9	PPAR signaling pathway	ACADM, ACSL5, CPT1B, GK, PPARA, RXRA, UCP1	6.6E−3	8.1E−2
10	Cardiac muscle contraction	COX5A, COX7A1, FXYD2, SLC9A1, UQCRC2, UQCRH	4.2E−2	2.9E−1

Abbreviations: ATP, adenosine triphosphate; CPT1B, carnitine palmitoyltransferase 1B; FDR, false discovery rate; KEGG, Kyoto Encyclopedia of Genes and Genomes; PPAR, peroxisome proliferator-activated receptor; TCA, tricarboxylic acid.

*P<0.05.
